# Beyond the bipolar: allowing satisfaction and dissatisfaction to coexist

**DOI:** 10.3389/fpsyg.2025.1572220

**Published:** 2025-10-10

**Authors:** Dustin J. Souders, Yixiu Yu

**Affiliations:** ^1^Department of Psychology, Clemson University, Clemson, SC, United States; ^2^Business Information Systems Department, Central Michigan University, Mount Pleasant, MI, United States

**Keywords:** satisfaction, dissatisfaction, ambivalence, indifference, bivariate unipolar items, bipolar scales

## Abstract

This paper advances a separable, multi-dimensional account of satisfaction and dissatisfaction, proposing that they are distinct constructs that can coexist, particularly in contexts involving ambivalence and indifference. When we specify measurement, we operationalize this account with bivariate unipolar items (separate single-ended scales for both satisfaction and dissatisfaction) rather than a single unidimensional bipolar continuum. Drawing upon theories from psychology—including Herzberg’s Two-Factor Theory, Watson and Tellegen’s Circumplex Model of Affect, Cacioppo’s Evaluative Space Model, and Reinforcement Sensitivity Theory—we argue that positive and negative emotions are processed separately in the human mind, allowing for the simultaneous experience of satisfaction and dissatisfaction, rather than placing them on two ends of a single bipolar spectrum. By reviewing evidence from psychological sciences, information science, human-computer interaction, and other disciplines, we highlight the limitations of bipolar scales in capturing the complexity of human emotional states and the full range of human experience. We review qualitative and empirical evidence demonstrating that satisfaction and dissatisfaction arise from different factors and have unique influences on behavior and decision-making. Recognizing them as separate constructs allows for more accurate measurement and better understanding of phenomena involving ambivalence and indifference. We offer practical guidance for researchers on when to use bivariate unipolar versus bipolar scales, how to identify contextual factors contributing to satisfaction and dissatisfaction, and how to design measurement tools that capture the nuances of these constructs. Through this narrative literature review, we embrace the idea that a separable, multidimensional perspective facilitates a more complete understanding of human emotions, motivations, and behaviors, leading to deeper insights and more effective strategies.

## Better understanding satisfaction and dissatisfaction: the cases of ambivalence and indifference

When we think about what makes us happy or frustrated in a given situation, we often simplify these emotions into two sides of the same coin–happiness on one side, frustration on the other. We perhaps feel like we could or should feel only one of these complex emotions, and when prompted, might skew our feedback in the direction of the stronger felt of the two. But does satisfaction entail the equal opposite of dissatisfaction in every situation? Does one unit of measurement of either contribute evenly in what amounts to a winner-takes-all tug of war between satisfaction and dissatisfaction? We argue in this paper that this is not the case when ambivalence (i.e., the simultaneous coexistence of conflicting attitudes, emotions, or evaluations toward a particular object, situation or decision) is present. Ambivalence represents a state where positive and negative emotions are felt at a near equal strength, leading to mixed or contradictory motivations that can affect behavior, decision-making, and emotional responses. There is a tension with ambivalence that does not exist with simple indecision or neutrality: it involves actively holding and balancing these opposing emotions and shaping how satisfaction and dissatisfaction are experienced and expressed. Dahl and Peltier (2015) review on over 30 years of consumer satisfaction research reflects this complexity, showing that consumer (dis)satisfaction cannot simply be understood as polar opposites. Rather, they identified multifaceted antecedents and outcomes, revealing the dynamic interplay that drives consumer behaviors and complaints beyond just simple measures of satisfaction. Building on this, Nowak et al. (2023) underscore the continued evolution of the field, particularly with the rise of brand love and dysfunctional consumer behaviors, which reflect emotions and actions that are both deeply positive and negative. Their findings suggest that the spectrum of consumer responses involves complex motivations and reactions that do not fit neatly within traditional bipolar scales of satisfaction.

Satisfaction has been studied from a consumer perspective (Dahl and Peltier, 2015; Nowak et al., 2023), with one’s job (Herzberg et al., 1959), or with one’s life (Davern and Cummins, 2006), but it is often oversimplified using a bipolar spectrum (Cacioppo et al., 1997; Howard and Sheth, 1969; Likert, 1932; Nicosia, 1966; Thurstone, 1931). This oversimplification does what many cognitive heuristics and biases do: it simplifies complex phenomena, allowing us to make timely and adaptive binary inferences based on incomplete or conflicting or otherwise imperfect information (Caverni et al., 1990; Haselton et al., 2015). Might the relationship between satisfaction and dissatisfaction be more subtle and complex than our cognitive biases and heuristics easily allow for? Indeed, Taylor (2008) expresses caution when trying to reconcile satisfaction and ambivalence for the purpose of modeling consumers’ judgment and decision making. A more holistic understanding through a bivariate unipolar lens allowing for coexistence should lead to more accurate measurement of these crucial motivational behaviors, as their shared and unique causes and effects can be quite more complex than poorly deployed bipolar Likert scales can capture (Schrum et al., 2023).

In this effort, we aim to support theory-based scientific inquiries that rely on accurately assessing phenomena as satisfactory or dissatisfactory by strengthening the concepts of satisfaction and dissatisfaction. Producing scholarship that lays the groundwork for robust concept formation, measurement development, and understanding causal relationships is crucial (Scheel et al., 2020). This robust concept formation is especially necessary when studying complex motivational attitudes that drive our approach and avoidance strategies in response to environmental stimuli that elicit satisfaction or dissatisfaction. Indeed, we forage for favorable ratios of satisfaction to dissatisfaction as we decide to persist in the current activity or choose to pursue another course of action to better meet our expectations. Our evaluations of both familiar and unfamiliar situations are limited by our sensory and cognitive systems and often arrive at states ranging from indifference (i.e., a state of emotional neutrality lacking in strong positive or negative feelings toward an object, situation, or decision) to ambivalence when the situation lacks clear valence. Indifference specifically denotes a lack of significant emotional engagement, motivation, or concern, leading to passive or disengaged responses.

The review portion of this manuscript will cite evidence from the Psychological Sciences, Information Science, Human-Computer Interaction (HCI), and other related disciplines to further question the traditional bipolar view of dis/satisfaction and help identify instances where it leads us astray. We draw on diverse literatures where observers came to one similar conclusion: that they were losing explanatory value by conceptualizing, measuring, and analyzing their phenomena of interest bipolarly. We continue by confirming and describing the separable, multidimensional view of dissatisfaction and satisfaction where they are separate and independent constructs instead of bipolar opposites.

## Satisfaction and dissatisfaction as separable unipolar constructs

Understanding the distinctions between satisfaction and dissatisfaction is crucial to grasping the full spectrum of human emotional experiences. While satisfaction is often defined as the fulfillment of one’s needs, desires, or expectations, dissatisfaction emerges when these same needs, desires, or expectations are unmet (Oliver, 2014). Although these two states are frequently seen as opposite ends of a continuum, the issue has remained unresolved for more than six decades (see Greenwald, 2012). This perspective has been increasingly challenged (Herzberg et al., 1959; Watson and Tellegen, 1985). The exploration of whether satisfaction and dissatisfaction are distinct or simply two sides of the same coin is vital for unraveling their roles in shaping our responses to various life situations, particularly when we decide to persist in an action or seek another route to satisfy these un/under-met needs, desires, or expectations. Traditional models have often fallen short of capturing the dynamic, non-linear nature of (dis)satisfaction interactions. Incorporating dynamical systems modeling allows for exploration of how these constructs evolve over time and interact with contextual factors, offering fresh insights into ambivalence and indifference. In this section, we will review the current advances regarding the two constructs and why and when we need to view them as two separate constructs.

Here, it is worth making a terminological note. In response to a well-placed readability concern from one of our reviewers, we describe our theoretical position as separable and multi-dimensional throughout the narrative. When we specify measurement and models, we use the technical term bivariate unipolar to denote that satisfaction and dissatisfaction are each assessed on their own single-ended continuum (from low/absent to high/present), rather than opposite ends of one scale. We reserve unidimensional solely for the rival bipolar conception that treats (dis)satisfaction as a single continuum; we use bipolar only in that sense.

A concrete illustration is helpful here. Imagine a food-delivery experience: the meal is outstanding (fresh, flavorful, exactly as ordered), but it arrives 45 min late. On separable, single-ended scales (i.e., bivariate unipolar), a customer might report high satisfaction with food quality (e.g., 9/10) and high dissatisfaction with timeliness (e.g., 8/10). A single bipolar rating would compress this into something near the midpoint, obscuring the occurrences of both praise and complaint. Treating satisfaction and dissatisfaction as separable, multi-dimensional constructs (operationalized with bivariate unipolar items) preserves that structure and yields distinct predictions: the satisfaction score better forecasts the customer’s willingness to recommend the restaurant and/or reorder from it, whereas the dissatisfaction score better forecasts complaint/attrition (e.g., refund requests or negative reviews). This is precisely the information that gets lost when the two are forced onto a single bipolar continuum.

### What is satisfaction? Is dissatisfaction something different?

Satisfaction is a multifaceted emotional state that we experience when our needs, desires, and expectations are met or exceeded (Oliver, 2014). It encompasses a sense of contentment, fulfillment, and well-being, often accompanied by positive emotions such as joy, pleasure, and gratitude. The satisfaction of an individual in each situation is influenced by numerous, complex factors that include the individual’s personal values (e.g., openness, loyalty), their past experiences, and their current situational context and its dynamics. In a technology use context, satisfaction generally reflects harmony between one’s expectations for a technological interaction and the reality of their actual experiences during that interaction (Bhattacherjee, 2001).

Often cast contrary to this, dissatisfaction is similarly a complex emotional state that occurs when an individual’s needs, desires, or expectations are *not* met, but dissatisfaction as a construct remains somewhat unclear (Souca, 2014). It involves feelings of frustration, disappointment, and discontent, and it often involves negative emotions such as anger, sadness, or resentment. Dissatisfaction can arise from a perceived imbalance between one’s goals and the achieved outcome, contributing to a sense of unfulfilled potential or unmet aspirations. Similar again to satisfaction, dissatisfaction is also impacted by personal values, past experiences, and the specific situational context. Again, putting it in a technology use context, dissatisfaction generally reflects deficits between one’s hopes for a technological interaction and the reality of their actual experience during that interaction.

Do these similarities mean that satisfaction and dissatisfaction are two sides of the same coin? Greenwald (2012) catalogues the structure-of-affect debate as one of psychology’s longest-running, still-unresolved controversies, underscoring the need for decisive empirical tests. Classic work by Bradburn (1969) already hinted that happiness and sadness can vary orthogonally, and research on mixed emotions shows that a strictly bipolar metric masks important nuance when people feel “bittersweet” blends of positivity and negativity (Thompson et al., 1995). As we will show, researchers from a variety of siloed fields have come to recognize the possibility of a bivariate unipolar view of the two, allowing for a dynamic interaction between them. This bivariate unipolar view is interactive and is opposed by a more fixed bipolar view that states satisfaction and dissatisfaction are the opposite ends of the same continuum, different sides of the same coin. It provides a more flexible view for understanding the complexities of ambivalence, seeing how satisfaction and dissatisfaction co-mingle to produce the range of our general experience when things are not clearly dissatisfying or satisfying.

In a broad literature review on (dis)satisfaction, Souca (2014) emphasized that dissatisfaction, satisfaction, as well as delight may each occupy distinct places along a continuum of affect. Souca’s findings suggest dissatisfaction does not simply stand as the opposite of satisfaction; rather, dissatisfaction is closer to the negative extreme while delight occupies the intense positive extreme, with satisfaction resting between these poles. The key distinction is the degree and intensity of the affective response: dissatisfaction tends to evoke more potent negative emotions (e.g., anger, frustration), whereas delight arises from unexpected, intensely positive experiences. This distinction underscores that measuring only “satisfied” vs. “not satisfied” can miss the richer interplay among dissatisfaction, satisfaction, and delight.

Building upon the notion that (dis)satisfaction are separable, multi-dimensional constructs, recent psychometric advancements offer valuable insights. Taking cues from recent psychometric work on non-cognitive constructs (Tay and Ng, 2018), an ideal point modeling (IPM) approach aligns well with our premises and provides a refined perspective on measuring satisfaction and dissatisfaction as distinct, yet interrelated, non-linear constructs rather than bipolar opposites. IPM posits that individuals are most likely to endorse items that closely match their own levels of the trait being measured—in this case, satisfaction and dissatisfaction. This approach captures the non-monotonic (inverted U-shaped) relationship between the latent trait and the probability of endorsement, allowing for the possibility that high levels of both satisfaction and dissatisfaction can coexist within the same individual. Importantly, IPM can operate with multiple dimensions, enabling satisfaction and dissatisfaction to be shaped by different situational factors while recognizing their potential to interact dynamically. By modeling satisfaction and dissatisfaction as separate but interacting dimensions, IPM aligns with a unipolar view, offering a more detailed and accurate reflection of complex emotional states.

Furthermore, by incorporating dynamical systems modeling—specifically using differential equations to capture behavior over time—we can model (dis)satisfaction as separate but dynamically interacting dimensions (see Richardson et al., 2014). This approach acknowledges that the interplay between (dis)satisfaction is nonlinear and can result in sudden shifts in emotional states when certain parameters reach critical thresholds, a phenomenon known as bifurcation in dynamical systems theory (Guckenheimer, 2011; Li et al., 2010). For example, models like the cusp catastrophe illustrate how minor changes in situational factors can lead to abrupt transitions between steady states, and this might also be true of satisfaction and dissatisfaction. By dynamically modeling these behaviors over time, we enhance the IPM approach, providing a framework that captures the complex, time-dependent interactions between satisfaction and dissatisfaction feedback loops. This aligns with the unipolar view and offers a more complete and accurate reflection of complex emotional states.

### Herzberg’s two-factor theory

**Figure 1:**
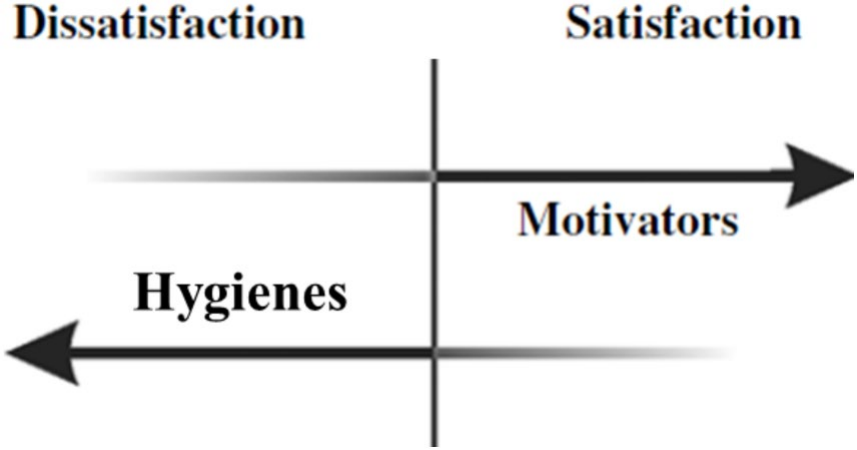
Herzberg’s motivators and hygienes.

One of the first widely cited sources to discuss unipolar constructs of satisfaction and dissatisfaction and the extent to which they interact is Herzberg’s two-factor theory (Herzberg et al., 1959), also known as Herzberg’s motivation-hygiene theory. Developed after analyzing interviews engineers and accountants, this theory suggests that job satisfaction and dissatisfaction are not merely opposite ends of a bipolar spectrum but are instead separate, distinct dimensions (Herzberg et al., 1959). According to the theory, two distinct sets of factors contribute to these dimensions: hygiene factors and motivation factors (Herzberg et al., 1959). Figure 1 visually represents motivators’ and hygienes’ differential contributions to (dis)satisfaction, which we expand upon below.

Hygiene factors such as salary and job security are linked to job dissatisfaction (Herzberg et al., 1959; Herzberg, 1966). When hygiene factors fall below a certain threshold, job dissatisfaction arises. However, even when these factors are optimized, they do little to add to job satisfaction—they merely prevent dissatisfaction. For instance, pay must be commensurate with the work performed to avoid dissatisfaction, but beyond a certain point, additional pay does little to enhance satisfaction.

On the other hand, motivation factors, such as responsibility, recognition, and participation in decision-making, are linked to job satisfaction. These factors must exceed a certain threshold to positively influence job satisfaction. For example, when employees are given meaningful opportunities to participate in decision-making processes, their sense of ownership and engagement increases, thereby enhancing job satisfaction. Unlike hygiene factors, motivation factors have the potential to truly enrich an employee’s work experience and foster a deeper sense of fulfillment.

## Psychological theoretical foundations of separability of satisfaction and dissatisfaction

Herzberg’s two-factor theory illustrates that satisfaction and dissatisfaction are distinct constructs rather than simply opposite ends of a spectrum. This distinction mirrors broader psychological frameworks that explore how positive and negative emotional experiences are processed separately in the human mind. By discussing psychological models like Watson and Tellegen’s Circumplex Model of Affect and Cacioppo’s Evaluative Space Model, we further expand this concept by demonstrating how ambivalent states with mixtures of positive and negative emotions can exist independently, providing a deeper understanding of complex emotional states that cannot be captured by a single bipolar continuum.

Building upon these models, we introduce additional theories that further support the unipolar view of satisfaction and dissatisfaction. Reinforcement Sensitivity Theory (RST) offers insight into how approach and avoidance motivations can be activated separately and simultaneously in response to stimuli (McNaughton and Gray, 2000). Additionally, the Metacognitive Model of Ambivalence (MCM) explains how individuals can hold conflicting evaluative beliefs about an attitude object, leading to ambivalence when they are aware of both positive and negative evaluations simultaneously (Song and Ewoldsen, 2015). This MCM emphasizes the role of metacognition in recognizing, managing, and responding to these conflicts through primary (automatic) and secondary (reflective) cognition and highlights how complex attitudes arise from the coexistence of positive and negative evaluations, reinforcing the idea that satisfaction are distinct constructs that can be experienced together.

As we integrate these theories, it becomes clear that a more differentiated understanding of satisfaction and dissatisfaction is required. This comprehensive perspective allows us to capture the full spectrum of human experiences, leading to more effective strategies for evaluating and enhancing well-being without overlooking potential sources of dissatisfaction.

### Watson and Tellegen’s circumplex model of affect

**Figure 2:**
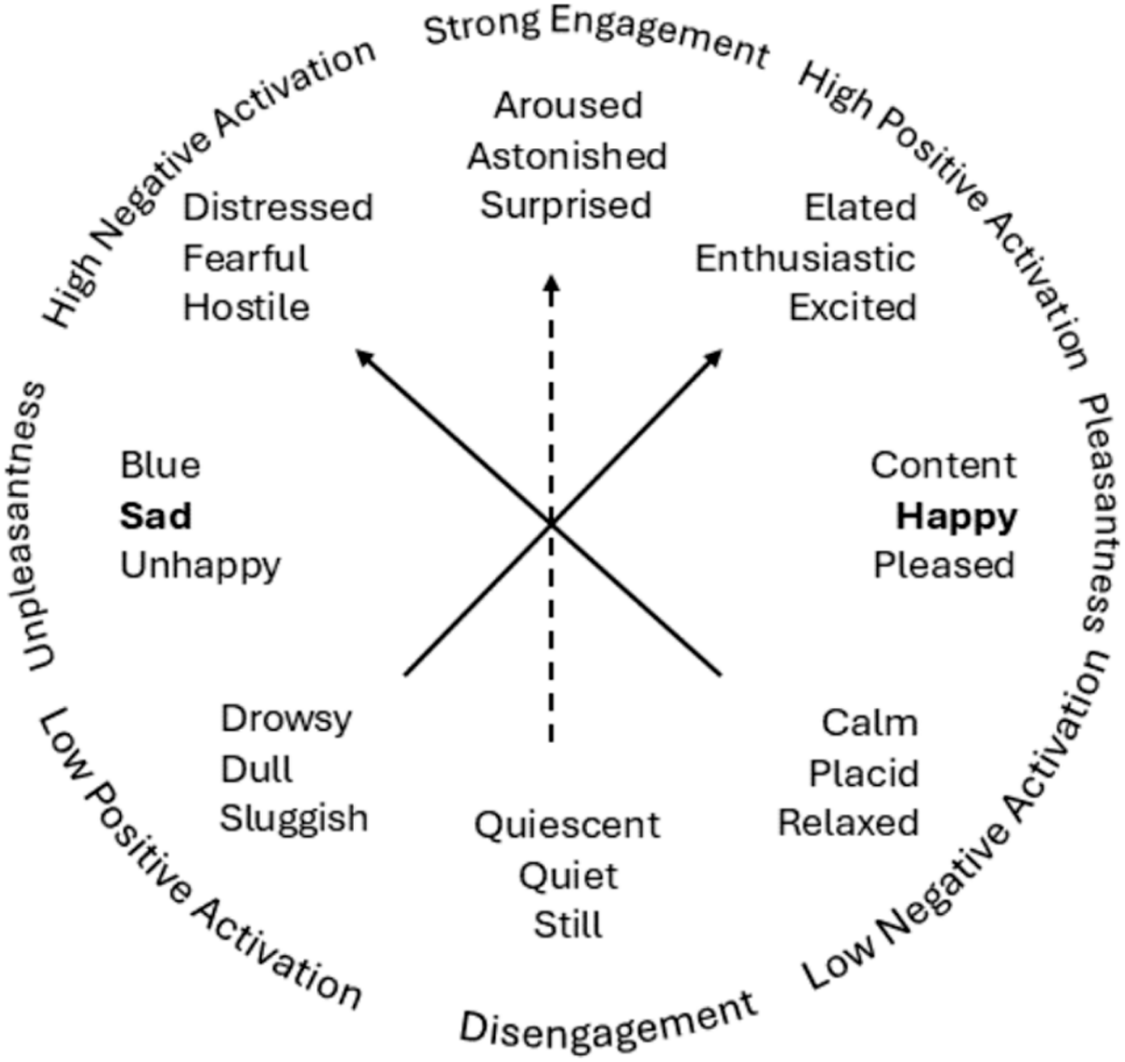
Watson and Tellegen’s circumplex model of affect with a dashed line for an engagement axis.

Foundational research by Watson and Tellegen (1985) shows that positive affect (PA) and negative affect (NA) are separable, allowing for complex emotions like bittersweetness (Larsen, 2017; Larsen et al., 2001; Man et al., 2017; Vaccaro et al., 2020). See Figure 2, where we have highlighted the engagement axis with a dashed line. The high ends of PA or NA represents a state of high affect emotional arousal; and the low end of PA or NA represents a relative absence of affective involvement (Watson and Tellegen, 1985). By plotting the dimension of engagement against the dimension of pleasantness, the circumplex model gives us a space where the amount of positive or negative activation can be charted and we can see that the opposite of PA is not NA but the absence of PA (Watson et al., 1999; Watson and Tellegen, 1985). That is, the presence of a positive affect does not preclude the presence of a negative affect (Watson et al., 1988; Watson and Tellegen, 1985).

The Positive Affect Negative Affect Scale (PANAS) further confirms the unipolar nature of PA and NA (Watson et al., 1988, 1999), and itself has been a reliable measurement tool of PA and NA for decades. Its further development with the PANAS-X (Clark and Watson, 1994) added seven dimensions to specific emotional states (Fear, Sadness, Guilty, Hostility, Shyness, Fatigue, and Surprise) as well as four positive affect dimensions (Joviality, Self-Assurance, Attentiveness, and Serenity), allowing for valid and reliable assessment of specific emotional states, traits, and affective experiences.

### Cacioppo’s evaluative space model

The distinguishable positive and negative affective processes are well supported by Cacioppo’s Evaluative Space Model (ESM) (Cacioppo et al., 1997; Cacioppo and Berntson, 1994) and its subsequent research (Cacioppo and Gardner, 1999; Larsen, 2017; Larsen et al., 2001, 2009, 2017, 2021; Norris et al., 2010). The ESM is a psychological framework that provides a comprehensive understanding of how humans process mixed emotions and challenges the view that positive and negative emotions exist on a singular bipolar continuum. Instead, ESM posits that positive and negative emotions are processed independently in the brain, allowing for the possibility of experiencing both types of emotions simultaneously. This allows attitudes to be better understood as bivariate space rather than a bipolar continuum that implies equivalence or interchangeability between approach and avoidance motivations (Cacioppo et al., 1997).

Crucially, the ESM introduces the concepts of *reciprocity* and *coactivity*. Reciprocity refers to how the activation of one emotion can influence the activation of another, exemplified by the mixed nature of emotions such as bittersweet memories. Coactivity, on the other hand, emphasizes the independent and simultaneous operation of positive and negative evaluations—allowing for scenarios where an individual may feel both positively and negatively about the same experience without one emotion negating the other (Cacioppo and Berntson, 1994, 1999). This is adaptively functional and allows for more flexible and contextually responsive behavior when situations are complex. For example, the joy of a promotion can also be tinged with the dread of increased responsibilities and allowing both states helps better explain the nervous excitement that might accompany such a role shift. This coactive nature aligns with theories that differentiate approach and avoidance as independent motivational systems.

By integrating ESM, we gain a richer understanding of mixed emotions in decision-making, particularly in situations characterized by ambivalence. For instance, when deciding whether to switch from one product or service to another, an individual might feel excited about the potential benefits of the new option while simultaneously feeling anxiety about leaving the familiar option behind. The ESM’s framework helps to quantify such ambivalence and has been applied in diverse studies, such as measuring electromyographic responses in gambling tasks (Larsen et al., 2009) and analyzing transportation mode choices using hybrid choice modeling (Borriello et al., 2019). Thus, the ESM not only aids in understanding the coexistence of positive and negative evaluations but also provides insights into evaluative mechanisms that guide complex behaviors in ambivalent contexts. The co-active nature of positive and negative evaluations aligns with theories that differentiate approach and avoidance as independent, rather than opposite, motivational systems which we describe next.

### Reinforcement sensitivity theory

While ESM focuses on emotional co-activation and reciprocation, theories of approach and avoidance, such as those grounded in reinforcement sensitivity theory (RST) (McNaughton and Gray, 2000) or gain/loss frameworks (Tversky and Kahneman, 1974), extend this understanding by showing how individuals can be simultaneously driven to pursue potential rewards and avoid potential losses in complex decision-making contexts. We next briefly describe the interplay between the behavioral activation system (BAS) and its approach response to rewarding stimuli, the behavioral inhibition system (BIS) and its avoidance response to aversive stimuli, and the fight-flight-freeze system (FFFS) that mediates responses to immediate threats.

#### Behavioral activation system

Being linked to positive emotions like excitement and anticipation, the BAS drives individuals toward desirable outcomes. It is thought to involve dopaminergic pathways and brain regions like the nucleus accumbens and ventral striatum (McNaughton and Gray, 2000). High BAS activity can lead to increased impulsivity and risk-taking, as the system encourages individuals to seek out rewarding stimuli (Alloy et al., 2006).

#### Behavioral inhibition system

Alternatively, the BIS regulates avoidance behavior and is sensitive to signals of punishment, uncertainty, or novel stimuli. It strives to resolve goal conflicts, such as when there is a choice between approaching a reward and avoiding a potential punishment. Linked to anxiety and cautious behavior, the BIS triggers arousal when threats or potential dangers are detected. Neurobiologically, the BIS is thought to involve the septo-hippocampal system, amygdala, and prefrontal cortex (Fuentes et al., 2012).

#### The fight-flight-freeze system

Finally, the FFFS mediates responses to immediate threats and danger, leading to aggression (fight), escape (flight), or immobilization (fear). It is highly sensitive to aversive stimuli and is tied to fear and panic responses. The FFFS involves brain structures like the amygdala and hypothalamus, which activate autonomic responses to danger (Graeff, 1994; Misslin, 2003). Therefore, the FFFS might be significantly activated by potentially dangerous stimuli and experiences (e.g., an automated vehicle strongly braking suddenly and unexpectedly).

#### The interplay of systems: heterarchical organization and navigating the complex balance

While the BAS, BIS, and FFFS provide a useful framework for understanding human behavior, it is important to recognize the complexity and potential overlap of these systems. The BIS and FFFS, which are involved in responding to aversive stimuli, are hard to distinguish between empirically and can be complex to interpret due to their shared underlying neural circuits and overlapping functions (Corr, 2016; Heym et al., 2008). Specifically, where they differ is the type of threat they address—BIS being more oriented to anxiety and conflict resolution while the FFFS is associated with immediate fear and escape responses. Both systems often coactivate and interact across different levels of neural processing. This is reflected in the complex interactions and recurrent processing that occur across the hierarchical levels of the way the brain processes information, where defensive strategies can shift rapidly between approach and avoidance (Norman et al., 2011), making it difficult to empirically separate their effects for measurement and interpretation purposes. As higher neural systems can inhibit or override lower-level substrates, they introduce further complexity into our understanding of these networks. Consequently, any simplified account of how these neural networks and cognitive systems interact must acknowledge the intricate and interconnected nature of these systems which collectively shape our behavior. As we continue, it is essential to consider these intricacies in our exploration of behavioral responses.

Recent research on RST demonstrates that approach driven by the BAS and avoidance driven by the BIS are activated separately and simultaneously in response to stimuli. For example, Warr et al. (2021) found that positive and negative affect align with approach and avoidance motivations, respectively, but these states can co-exist. Further, research in career decision-making has found that BAS sensitivity (associated with reward and satisfaction) and BIS sensitivity (associated with avoidance and dissatisfaction) predict different motivational dispositions in career planning (Corr and Mutinelli, 2017). This dual activation shows that feelings of excitement and anxiety can co-exist in complex decision-making scenarios. While RST focuses on motivational systems, we next explore a metacognitive processes in managing conflicting evaluations.

### Metacognitive model of ambivalence

The metacognitive model (MCM) of ambivalence explains ambivalence as arising from conflicting evaluative beliefs about an attitude object (Song and Ewoldsen, 2015). It emphasizes the role of metacognition in recognizing, managing, and responding to these conflicts. According to the MCM, individuals experience ambivalence when they are aware of holding positive and negative evaluations simultaneously. According to the MCM, this can be driven by both subjective ambivalence (e.g., the feeling of internal conflict) and objective ambivalence (e.g., a structural feature of attitudes with conflicting positive and negative evaluations). These competing evaluations of an attitude object may not always correlate with each other (Priester and Petty, 1996).

Both primary and secondary cognition play crucial roles in forming and managing ambivalence. Primary cognition refers to the initial evaluative judgments about the attitude object. These are often automatic and unconscious, tied to attributes such as positive or negative feelings about the object. Secondary cognition, however, involves a more reflective process. It acts as a metacognitive layer that evaluates the validity or confidence in the primary cognition. For instance, one might consciously reconsider whether a product’s affordability really makes it a good choice, potentially tagging the initial positive evaluation as invalid if new information suggests the product is of low quality. This more deliberate judgment can magnify or suppress the influence of primary cognition on one’s overall attitude.

The interaction between these two forms of cognition allows for ambivalence, as both positive and negative primary evaluations can coexist, moderated by secondary cognition. This subtle mechanism helps explain why people can experience mixed emotions or conflicting attitudes toward the same object or situation.

As we transition to a deeper exploration of satisfaction and dissatisfaction’s ability to co-exist, it becomes clear that a better understanding of these mixed emotional states is required. The unipolar view offers this by acknowledging that satisfaction and dissatisfaction are not merely opposites on a bipolar scale but independent and co-existing phenomena, much like the separability of positive and negative emotions in models like Watson & Tellegen’s Circumplex Model and Cacioppo’s ESM. RST complements this discussion by highlighting how individual differences in sensitivity to reward and punishment can shape emotional experiences, including ambivalence. Individuals with high reinforcement sensitivity may experience greater fluctuations between satisfaction and dissatisfaction due to their heightened responsiveness to positive and negative feedback in their environments (Smederevac et al., 2014). This sensitivity can influence how they interpret and manage mixed emotions, potentially leading to a more nuanced understanding of their emotional states and the strategies they employ to navigate them (Lopez-Vergara et al., 2012). The MCM of ambivalence further enhances this understanding by emphasizing how individuals can reflect on and manage conflicting evaluations through metacognition, allowing both satisfaction and dissatisfaction to coexist consciously. In the same way that these models explain the coexistence of positive and negative affective states, unipolar interpretations of satisfaction and dissatisfaction allow us to capture the full complexity of human experiences. This view broadens our ability to evaluate and measure these states individually, leading to more effective strategies for improving satisfaction without overlooking potential sources of dissatisfaction.

## Unipolar satisfaction and dissatisfaction in various domains

This section provides a narrative review of key areas where researchers across disciplines have acknowledged the value of unipolar satisfaction and dissatisfaction constructs. It synthesizes extensive qualitative studies and emerging empirical research across topics such as human-computer interaction (HCI), psychological well-being, consumer behavior, education, technology adoption, and artificial intelligence (AI). Despite the diversity of these fields and the limited volume of existing studies, our review demonstrates how the unipolar view more accurately reflects the complex and layered nature of human evaluative processes. By accounting for asymmetric triggers and their diverse consequences, the unipolar perspective provides a more nuanced framework for understanding satisfaction and dissatisfaction as distinct yet interrelated phenomena.

### Insights from qualitative literature

Herzberg’s research and subsequent studies provide substantial qualitative evidence supporting the separability of satisfaction and dissatisfaction, particularly in technology and HCI contexts. The main methods are critical incident technique and text-mining approach.

#### Critical incident technique and hygiene vs. motivation factors

Qualitative evidence based on critical incident technique supports the separability of (dis)satisfaction dimensions, which result from distinct sets of hygiene and motivation factors. For example, in the context of smartphones, technical quality is a hygiene factor that must be performed well to prevent dissatisfaction (Tuch and Hornbæk, 2015). In contrast, motivation factors such as utility and convenience contribute to increased satisfaction (Tuch and Hornbæk, 2015). Regarding website UI features of websites, dissatisfaction arises when privacy and security are poorly managed, whereas user empowerment is essential for enhancing satisfaction (Zhang and von Dran, 2000). Additionally, a study on learning management systems found that satisfaction for both students and educators is influenced by a combination of environmental and job-specific factors, while dissatisfaction is driven solely by environmental factors (Islam, 2014). In the context of officer live streaming, researchers identified hygiene factors contributing to dissatisfaction, such as officer live streaming itself, product authenticity, officer streamer trustworthiness, and government credibility. On the other hand, motivation factors that lead to satisfaction include the officer streamer’s physical attractiveness, interaction friendliness, altruism, expertise, product price attractiveness, and the local uniqueness of the product (He et al., 2022). In similar research on users’ attitudes toward mobile fitness apps, dissatisfaction typically arises from issues related to functional features, compatibility, and paid services, while satisfaction is more closely associated with gratification, self-monitoring, and self-regulation (Kim and Lee, 2023).

#### Text mining and NLP-based approaches

Recent studies have built on Herzberg’s theory, applying natural language processing and machine learning to analyze large sets of textual data, such as online reviews, through text-mining techniques. For example, using topic modeling, researcher identified different sources of satisfaction and dissatisfaction in Airbnb accommodations (Ding et al., 2021). Using latent semantic analysis (LSA), another study revealed the determinants that lead to hotel customer satisfaction and dissatisfaction are different and are specific to particular types of hotels, including full-service hotels, limited-service hotels, suite hotels with food and beverage, and suite hotels without food and beverage (Xu and Li, 2016). Some studies integrate text analytics with fuzzy-set Qualitative Comparative Analysis (fsQCA) to explore casual recipes of factors influencing satisfaction and dissatisfaction. Lee (2022) identified service-dimension configurations that lead to guest satisfaction and dissatisfaction with accommodation sharing. Similarly, researchers have found different configurations of factors contributing to guest satisfaction and dissatisfaction and suggested hosts focus on key dimensions (order winners) that most affect customer loyalty (Lee et al., 2024). In the smart home context, researchers have found that various UX dimensions influence user satisfaction and dissatisfaction with smart home products (Yu et al., 2024). For example, in the case of robotic vacuums, key dimensions contributing to satisfaction include “functionality,” “smartness,” and “enhanced capacity (e.g., a mopping feature).” Conversely, “insufficient smartness,” “poor customer service,” and “connectivity problems” are critical dimensions of user dissatisfaction. Notably, “smartness” serves as a double-edged sword, potentially leading to high satisfaction but also significant dissatisfaction if poorly implemented. These studies extend Herzberg’s theory by identifying the differentiated sources of satisfaction and dissatisfaction.

### Insights from empirical studies

Empirical research underscores the necessity measuring (dis)satisfaction separately to accurately capture the complexity of human experiences. This section reviews key studies in psychological well-being, consumer behavior, and technology adoption that contribute to a more differentiated understanding of these constructs as separate, yet interrelated phenomena.

#### Psychological well-being

Studies in psychological well-being have increasingly challenged traditional bipolar models, which assume that satisfaction and dissatisfaction exist as two ends of the same continuum. Davern and Cummins (2006) demonstrated through cross-sectional survey data that life satisfaction and dissatisfaction are distinct constructs influenced by different variables. They proposed using unipolar scales to better reflect these dimensions, as bipolar scales may obscure their independent characteristics.

Mazaheri and Theuns (2009) further supported this perspective by investigating the efficacy of unipolar versus bipolar scales in capturing self-assessed life satisfaction among students. They found that unipolar scales allowed for more critical responses, highlighting that individuals often struggle to express dissatisfaction on bipolar scales. Boes and Winkelmann's (2010) analysis of income’s effects revealed an asymmetry: while income effectively reduced dissatisfaction, its impact on high satisfaction levels was minimal. This further underscored the independence of these constructs.

Similarly, Westerhof and Keyes’s (2010) Two Continua Model (TCM) argues that mental health and mental illness are distinct but interrelated. Their cross-sectional survey of Dutch adults found that older individuals experienced fewer symptoms of mental illness but did not necessarily exhibit higher levels of flourishing. This suggests that mental health and illness are distinct yet interrelated dimensions, much like (dis)satisfaction.

This perspective has practical implications: measuring and addressing satisfaction and dissatisfaction requires tailored interventions. As Morrison et al. (2023) observed, enhancing well-being often demands different strategies than reducing ill-being, supporting a dual-lens approach to psychological evaluation and intervention design. Their study, grounded in the dual continua model, tracked 351 students across six months, revealing that changes in well-being and ill-being were only weakly correlated (R^2^ = 0.214), defying the bipolar assumption. Notably, while many students experienced declining well-being, their levels of psychological distress (ill-being) did not necessarily increase. This divergence emphasizes that the two dimensions respond independently to external influences such as financial stability and physical health. Furthermore, Morrison et al. also employed latent profile analysis and identified six distinct mental health clusters, ranging from “flourishing” (high well-being, low ill-being) to “languishing” (low well-being and high ill-being). The longitudinal design allowed tracking dynamic transitions between these clusters. Students’ responses revealed complex pathways: some improved in both dimensions, others experienced gains in one but not the other, while still others saw declines in both. This supports the dual continua model’s assertion that mental health cannot be reduced to a single bipolar spectrum.

In parallel, Positive Psychology frameworks explore the dual nature of well-being and ill-being. Zhao and Tay (2023, 2024) examined the bipolar view, asserting that well-being and ill-being could be placed on a single continuum. Importantly, Zhao and Tay state that bipolarity does not equate to a simplistic binary framework where well-being is the mere absence of ill-being. Instead, they exist on a continuum, where reducing ill-being does not automatically result in higher well-being. They further argue that co-endorsements are methodologically acceptable as well, as individuals can simultaneously exhibit moderate levels of both without invalidating the bipolar framework, which contradicts the older notion that well-being and ill-being are mutually exclusive. Their use of unfolding item response models suggests that individuals can report well-being and ill-being simultaneously at different degrees, aligning with the bipolar conceptualization while allowing for complex response patterns (Zhao and Tay, 2024). However, bivariate research, such as Iasiello et al. (2024), posits that these dimensions are better understood as independent. Their findings suggest that interventions targeting well-being (e.g., gratitude exercises) might not reduce ill-being (e.g., anxiety). This decoupling mirrors the coexistence model proposed by ESM, which acknowledges simultaneous positive and negative experiences.

Collectively, these studies highlight the value of adopting a dual-framework perspective in understanding emotional and psychological states. Recognizing satisfaction and dissatisfaction as distinct but interrelated constructs offers a richer, more comprehensive view of human experience and its measurement. While this bipolar/bivariate debate continues in Positive Psychology, Zhao and Tay present compelling evidence for a bipolar view, but it is worth noting that the bivariate approach is more conceptually flexible and may offer deeper theoretical insights into distinct interventions and predictive models.

#### Consumer behavior

The separability of positive and negative customer experiences, i.e., satisfaction and dissatisfaction is further supported in research on consumer behavior. Babin and Griffin (1998) proposed that customer satisfaction and dissatisfaction are distinct emotional states. Similarly, Alegre and Garau (2010) argued that using the bipolar approach is insufficient to understand tourists’ attitudes toward destinations. Their study on sun and sand destinations revealed that tourist satisfaction and dissatisfaction indeed coexist, and negative experiences do not necessarily diminish overall satisfaction. They emphasized the need to consider both positive and negative aspects to obtain a comprehensive understanding of tourist experiences so that negative aspects can be addressed, and positive aspects can be enhanced. Acknowledging these differential contributions to dissatisfaction and satisfaction coexisted in the overall tourist experience and querying them separately gave clearer and more actionable insights.

Treating dissatisfaction as a distinct construct allows researchers to identify different service quality dimensions that lead to discontent and trigger positive versus negative consumer reactions (Khalek et al., 2025). For example, satisfaction can promote loyalty while dissatisfaction lead to consumer complaining behavior and negative word-of-mouth (Khalek et al., 2025). Notably, in their follow-up review, Nowak et al. (2023) observed a decrease in interest in consumer satisfaction methods a measurement topics from 2015 to 2022. They advocated to revitalize research foci in this area, especially given emerging constructs involving ambivalence, such as brand love and dysfunctional customer behaviors, and the rise in qualitative research methods.

Across these studies, a consistent thread emerges: dissatisfaction is not simply the absence of satisfaction but rather a distinct construct that can coexist alongside positive emotional states and provides separate information. Recognizing the importance of unipolar measures enables more accurate and actionable insights into consumer experiences, extending beyond the “either-or” perspective. Moreover, recent recommendations underscore the value of longitudinal and real-time approaches to capture the dynamic evolution of consumer affect. This evolving understanding of consumer satisfaction and dissatisfaction aligns with the calls by Nowak et al. (2023) for renewed focus on innovative measurement methods, ensuring that both researchers and practitioners can gain a more comprehensive view of what drives, sustains, and erodes consumer satisfaction in today’s complex marketplace.

#### Technology adoption

Similar to consumer behavior research, adopting a unipolar view of satisfaction and dissatisfaction clarifies how distinct positive and negative factors drive technology use (Yu and Davis, 2024). This perspective distinguishes satisfaction and dissatisfaction as independent constructs, enabling the identification of distinct pathways through which positive and negative antecedent factors influence each dimension (Yu and Davis, 2024). This unipolar framework is particularly useful in addressing the complexities of technology adoption and use. By separating positive and negative evaluations, they gained more refined insights into users’ intentions to continue using technology, reflecting the complexity of modern user experiences.

Further research has found that users separately appraise benefits (approach factors) and drawbacks (avoidance factors) of technology use, forming two parallel affective evaluations: satisfaction and dissatisfaction (Yu and Davis, 2025). These evaluations are then jointly considered when making post-adoption decisions regarding continuance, discontinuance, or switching. Empirical evidence both U. S. and German samples supports this dual-evaluation mechanism.

Um et al. (2021) illustrated this independence by revealing the asymmetry between the impacts of satisfaction and dissatisfaction on continuance intentions in blended learning courses. Their classification of service dimensions as delighters, satisfiers, and dissatisfiers showed that satisfaction significantly boosts continuance intention, whereas dissatisfaction negatively impacts it to a lesser, though still meaningful, extent. Similarly, Bortne et al. (2024) adopted a unipolar approach to examine the relationships between beliefs, emotions, and bank-switching intentions and behaviors. Their study highlighted the multifaceted nature of contemporary technology, especially in the context of emerging AI-powered products.

In sum, these studies highlight the value of unipolar satisfaction and dissatisfaction in several ways: (1) Unipolar models better account for these asymmetric triggers and their diverse consequences, (2) they more accurately reflect the complexity of human evaluative processes, especially in the ambivalence associated with evaluation, and (3) they clarify intensity and salience by making it clearer how both satisfaction and dissatisfaction uniquely influences behavior—something critical to understand and predict user responses.

Looking beyond technology use, empirical evidence across diverse fields supports the separability of (dis)satisfaction. By adopting a unipolar approach, research can more accurately capture the complexity of human experiences, paving the way for enhanced understanding and better-informed strategies in psychological well-being, consumer behavior, and technology adoption. However, an important gap in existing literature is the lack of dynamic, longitudinal measurement. Such an approach could illuminate how satisfaction and dissatisfaction evolve and interact over time, revealing crucial turning points, feedback mechanisms, and patterns of stability or change. Integrating these dynamic perspectives would not only fill a significant gap in the research but also provide a richer framework for predicting and influencing outcomes across a variety of domains.

### Empirical resolution: testing the separability of satisfaction and dissatisfaction

This section moves beyond conceptual arguments and provides direct statistical evidence that satisfaction and dissatisfaction function as separable constructs rather than simply opposite poles of a single bipolar construct.

To evaluate their separability, we conducted correlational, factor-analytic, and incremental validity tests using the dataset from Yu and Davis (2024), which included post-adoption technology users (*N* = 214). The dataset contains four constructs: unipolar satisfaction and unipolar dissatisfaction (each measured on a 5-point scale from 0 = not at all to 4 = extremely satisfied/extremely dissatisfied, respectively), bipolar satisfaction (measured on a 9-point scale from 1 = extremely dissatisfied to 9 = extremely satisfied, with 5 = neutral), positive expectation disconfirmation (0 = not at all to 4 = extremely), negative expectation disconfirmation (0 = not at all, 4 = extremely), and continuance intention (measured on a 7-point scale from 1 = strongly disagree to 7 = strongly agree).

#### Measurement model comparison

We compared a two-factor model, in which satisfaction and dissatisfaction were modeled as distinct latent variables, with a one-factor model, in which all items loaded on a single latent factor. Statistically, the one-factor model fit the data better (χ^2^ = 36.32, df = 8, CFI = 0.96) than the two-factor model (χ^2^ = 156.43, df = 25, CFI = 0.90). While these fit indices suggest some empirical overlap, several other indicators support the two-factor conceptualization. The correlation between the latent constructs was moderate (*r* = −0.48), discriminant validity criteria were met (HTMT = 0.55 < 0.85), and the constructs displayed distinct nomological patterns. As shown in Table 1, satisfaction correlated positively with positive disconfirmation (*r* = 0.47, *p* < 0.001) and continuance intention (*r* = 0.57, p < 0.001), whereas dissatisfaction correlated positively with negative disconfirmation (*r* = 0.33, p < 0.001) and showed weaker, often inverse, associations with the other constructs. Taken together, these results indicate that although the one-factor model offers a more parsimonious statistical fit, the two-factor specification is theoretically meaningful and empirically supported when considering discriminant validity, differential antecedents, and incremental predictive utility.

**Table 1:** Correlations of other construct with user satisfaction and dissatisfaction.

Outcome variable	Satisfaction	Dissatisfaction
Positive expectation disconfirmation	0.47***	−0.22***
Negative expectation disconfirmation	−0.27***	0.33***
Continuance Intention	0.57***	−0.45**

#### Predictive validity

We further examined whether treating satisfaction and dissatisfaction separately provided predictive advantages for continuance intention compared to using a combined bipolar score. In hierarchical regression, adding the two separate predictors significantly increased explained variance by 5% (ΔR^2^ = 0.05, *p* < 0.001) over the combined measure. This finding underscores the practical utility of the unipolar framework for modeling post-adoption behavior, even when measurement-level fit indices favor a parsimonious one-factor specification.

In sum, while our measurement model comparison revealed that a single-factor specification offered a marginally better statistical fit, the balance of evidence, from discriminant validity indices to distinct nomological networks and incremental predictive power, supports the treatment of satisfaction and dissatisfaction as separable, unipolar constructs. This distinction is not merely theoretical; it provides greater diagnosticity, enabling the identification of factors that uniquely drive positive versus negative evaluations. These findings lay the empirical groundwork for the discussion that follows, in which we situate our results within a broader methodological and theoretical context, examine the limitations of bipolar measures, and outline the advantages of adopting a bivariate unipolar framework for capturing the complexities of ambivalent user experiences.

## Discussion

Recent research emphasizes that psychologists cannot assume seeming opposites, like (dis)satisfaction, lie on a single bipolar continuum (Barchard et al., 2024). As Scheel et al. (2020) stress, we seek to strengthen the “derivation chain” linking theory to empirical outcomes by refining the definitions of satisfaction and dissatisfaction, proposing improved measurement techniques, and identifying contextual nuances, we seek to construct a more robust foundation upon which future confirmatory studies measuring satisfaction can rest.

However, as Bargheer (2025) emphasizes, the popularity of the Likert scale and its longstanding forced reliance on unidimensional, bipolar scales stems not merely from theoretical conviction, but from the historical bundling of theory, method, and prototypical cases. This “package-deal” fosters ease of application and familiarity for researchers and participants alike, even though it may obscure ambivalent states where positive and negative evaluations coexist. In the latter parts of this paper we strive to differentiate the “package-deal” of bivariate unipolar interpretations of (dis)satisfaction.

In keeping with this perspective, Höhne et al. (2022) highlight the limitations of bipolar scales in capturing the complexity of attitudinal evaluations particularly in contexts like income inequality. These scales can compress opposing sentiments into a single dimension, reducing clarity and overlooking the coexistence of distinct states (e.g., contentment and discontentment). Adopting a unipolar perspective acknowledges that satisfaction and dissatisfaction are not mere opposites but can be influenced by different factors and coexist in the same context. This approach accommodates ambivalence and enables more accurate reflection of respondents’ experiences, thereby supporting more targeted strategies to enhance satisfaction and address dissatisfaction.

For a measurement to be valid, it must be both selective and diagnostic (Swets, 1988). Ambivalence—where positive and negative feelings coexist (e.g., satisfaction with reliability but dissatisfaction with transparency)—challenges both criteria. Selectivity declines as single, bipolar measures struggle to isolate specific elements being evaluated, often conflating overlapping or conflicting emotions (Moore, 1973). This obscures *which* aspects are driving changes in, for instance, overall satisfaction. If someone is “somewhat dissatisfied,” is it the functionality that upsets them, the usability, or something else?

Recent research (Barchard et al., 2024) about bipolar vs. bivariate measurement dovetails with this: if two constructs are genuinely opposite ends of a single continuum, a bipolar scale can be justified, *provided* that extreme positive always excludes extreme negative. However, ambivalent states—where positive and negative can each be substantial—reveal gaps in such bipolar measures. The conflate or mask the root causes of (dis)satisfaction, undermining diagnosticity (Mattson et al., 2013).

### Nonlinear and dynamic perspectives on (dis)satisfaction

#### Accommodating non-monotonic effects with IPM

Capturing the varying strengths of these opposing influences is further complicated by the non-linear interplay between (dis)satisfaction. Incorporating IPM, as discussed by Tay and Ng (2018), addresses these issues by accommodating non-monotonic relationships, allowing for a more sophisticated understanding of how individuals respond to items that align with their latent trait levels. This approach effectively handles the coexistence of positive and negative feelings when ambivalence is high, as the nonlinearity grows more pronounced because conflicting influences push in different directions. Barchard and colleauges’ (2024) discussion of censored data models (i.e., partial measurement of each end of a continuum) aligns with the idea that (dis)satisfaction might each be triggered by different, sometimes unrelated, factors. IPM is a methodological strategy that can flexibly handle these complexities, something basic bipolar measures often cannot.

#### Time-based dynamical analysis

Bipolar models traditionally measure a single dimension at one snapshot in time. Yet ambivalence is *dynamic*: a user may be satisfied with design at one moment and simultaneously dissatisfied with new pop-up ads introduced in the next. Incorporating time-based dynamical analysis methods (Castell and Schrenk, 2020) can further enhance diagnosticity by *tracking* shifts in (dis)satisfaction over time, revealing transitional states and the identifying the drivers that trigger these changes. Consistent with Barchard et al.' (2024) emphasis on censored data analysis (where each end of the continuum is measured), time-based approaches can reveal *when* positivity and negativity co-occur or dominate, helping pinpoint *what* triggers these transitions. This supports stronger diagnosticity by capturing fluctuations rather than a single “net” state.

Together, these methods offer tools to navigate the complexity of ambivalence, providing deeper insights into the non-linear interactions at play. By acknowledging that (dis)satisfaction are dynamic processes influenced by various factors, we pave the way for innovative methodological approaches. Dynamical systems modeling, introduced earlier, offers a valuable framework for visualizing and analyzing these temporal patterns and feedback loops.

Despite these challenges, a unipolar approach—treating satisfaction and dissatisfaction as distinct and independent constructs—provides a path forward. This framework increases both selectivity and diagnosticity by enabling researchers to account for context-specific subtleties. By identifying satisfying and dissatisfying factors, assisting their relative potency and interactions, and accommodating asymmetries, researchers can better measure and interpret satisfaction in ambivalent scenarios. The remainder of this paper offers practical guidance for applying these principles to improve measurement accuracy and address the challenges posed by ambivalence.

### Unipolar bivariate approaches for ambivalence

#### Selecting bipolar vs. unipolar bivariate measures

When a bullet is shot from a gun, it is of course important to know where its position is, but the more we focus on position the more we lose track of other variables of importance (e.g., its velocity). In a similar way, if we rely solely on a single bipolar scale—where satisfaction and dissatisfaction trade off directly—we know whether someone was on-the-whole satisfied or dissatisfied, but we overlook the more nuanced details of someone’s experience. Sometimes a quick, high-level snapshot (like the position of the bullet) is all that is needed, but other times we require finer granularity (like the velocity of the bullet) to capture the full picture of its trajectory.

**Figure 3:**
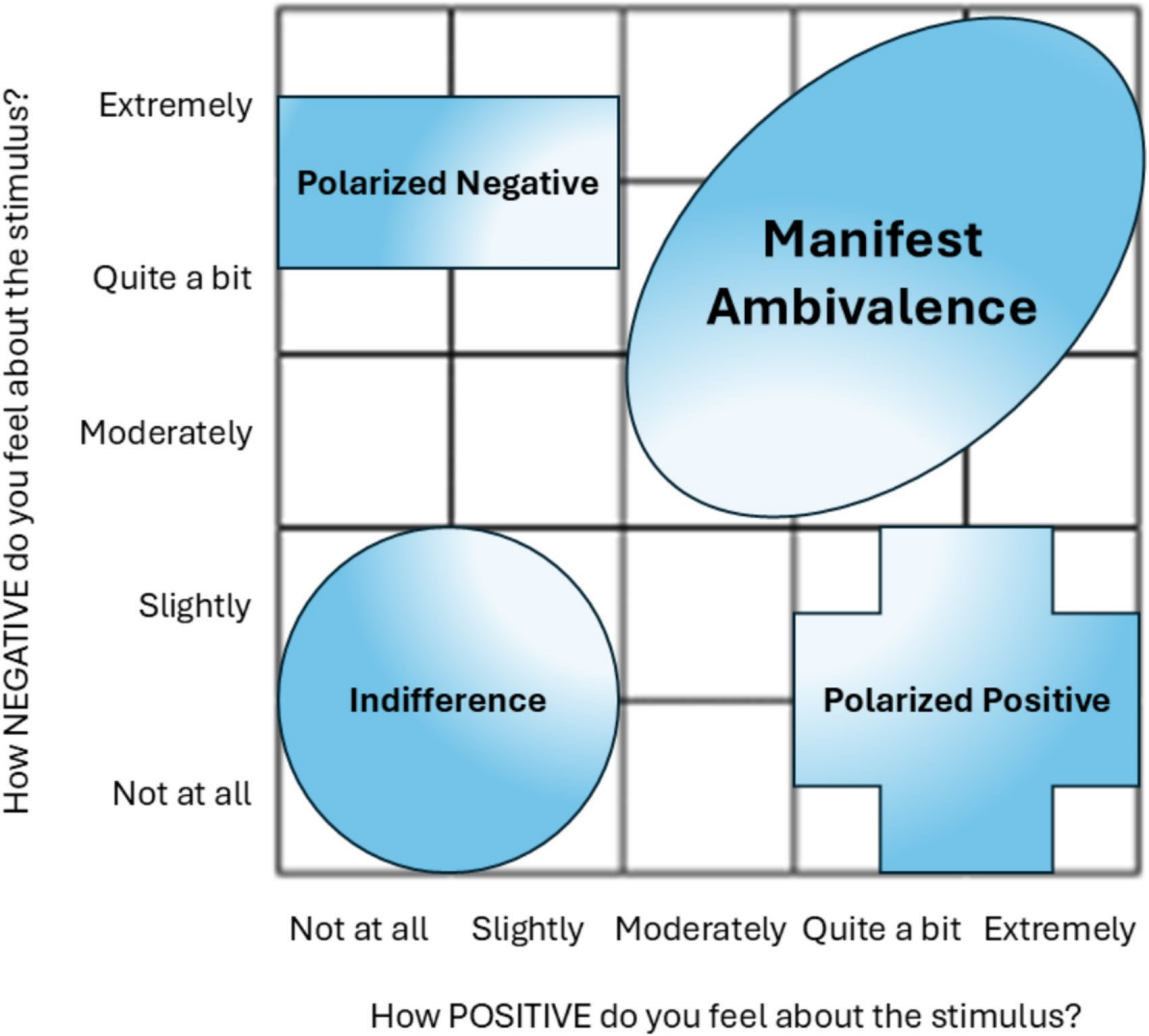
Adapted from Audrezet and Parguel (2018). Interpretation of the different zones of the ESG. Gradients represent changes in strength.

If the research question only ponders *whether* people feel satisfied or dissatisfied, a single bipolar scale works. It is straightforward and captures the “headline” of satisfaction vs. dissatisfaction. This is like taking a quick snapshot (position) or a simplified measure (velocity) without all the details in between. Bipolar scales are especially effective when attitudes are stable and predominantly positive or negative. Stable, high levels of positive and negative activation on the Evaluative Space Grid (ESG; see Figure 3), can justify the use of bipolar scales when attitudes are strong and univalent (Audrezet and Parguel, 2018; Larsen et al., 2009). For example, during product use, users who experience pure satisfaction or dissatisfaction can easily report their feelings using bipolar scales, as demonstrated in studies like Yu and Davis (2024). In these scenarios, bipolar measures are parsimonious and sufficient for capturing univalent emotions.

While bipolar measures have their place, particularly when satisfaction and dissatisfaction trade off at equal rates, we argue that they are not always the correct measurement tool. If the research question ponders *why* or *how* people feel satisfied or dissatisfied, which often involves teasing apart different dimensions of satisfaction and dissatisfaction, then separate unipolar measures for each dimension are more appropriate. By measuring both separately, you are essentially preserving more “velocity data” through finer granularity that avoids “compressing” everything into a single continuum. By allowing dissatisfaction and satisfaction to co-mingle, it allows for an ambivalence index (Herr et al., 2022) that creates local maxima (ambivalence) and minima (polarized stable alternative states).

#### Differentiating ambivalence and indifference

Ambivalence, where positive and negative perceptions coexist, calls for unipolar measures. Cognitive dissonance theory (Festinger, 1962) and decision-making theories (Janis and Mann, 1977), emphasize that mixed feelings are common. For example, a user may appreciate a website’s responsiveness while disliking intrusive advertisements (Cenfetelli, 2004). Such conflicting perceptions require unipolar measures to capture the complexity of emotions and avoid oversimplification inherent in bipolar scales. Humans commonly juggle multiple disparate and conflicting perceptions when evaluating a target and subsequently arriving at an attitude and a choice (Bettman et al., 1998).

Ambivalence is most pronounced when competing evaluations are intense and similar in magnitude, as shown in cell (5,5) of the ESG (Larsen et al., 2009). This state is highly volatile and susceptible to biases like loss aversion, where negative experience outweigh positive ones (McGraw et al., 2008). Unipolar measures allow for detailed insights into the mechanisms of ambivalence, such as trade-offs between expected benefits and anticipated costs.

Unlike ambivalence, which involves strong but conflicting evaluations, indifference reflects a lack of strong feelings in either direction. The mid-point on bipolar scales often fails to distinguish between these states, leading to misinterpretation (Thompson et al., 1995). Some respondents actually report they held mixed feelings though they selected the middle point on the bipolar scale (Yu and Davis, 2024). Unipolar measures better capture the idiosyncrasies of both ambivalence and indifference (see Table 2).

**Table 2:** ESG Implications for bipolar/unipolar distinctions.

Valence	Polarity	Example	Why	Models that align
High positive	Bipolar	Product quality	High uniformity of positive or negative collective feeling allows for strong bipolar interpretation	Expectancy-disconfirmation theory, Kano model
High negative	Bipolar	Product quality	High uniformity of positive or negative collective feeling allows for strong bipolar interpretation	Expectancy-Disconfirmation Theory, Kano model
High ambivalence	Unipolar	Satisfaction, trust	More co-mingling of positive and negative factors, varying level of their importance and impact	Herzberg’s two-factor theory, resilience theory, cognitive dissonance theory
High indifference	Unipolar	Employee benefits	Indifference often arises from a lack of strong feelings either way, leading to a situation where positive and negative factors do not strongly influence each other	Herzberg’s two-factor theory, control theory

### Identification of contextual factors contributing to satisfaction and dissatisfaction using qualitative techniques

Effectively measuring (dis)satisfaction begins with identifying the contextual factors that shape these constructs. Qualitative methods, such as the Critical Incident Technique (CIT), text-mining, and Hierarchical Value Mapping (HVM), are essential for capturing the varied dynamics of unipolar constructs like (dis)satisfaction. CIT, for instance, helps identify key factors that drive (dis)satisfaction by analyzing specific user experiences. In human-computer interaction research, privacy concerns often emerge as dissatisfaction drivers, while features like user empowerment can serve as motivators when implemented effectively (Zhang and von Dran, 2000). Similarly, text-mining techniques provide a way to uncover hidden drivers by analyzing large datasets, such as user-generated reviews, revealing subtle influences that might otherwise go unnoticed. Ding et al. (2021) used Airbnb reviews to find hidden drivers of satisfaction and dissatisfaction through analyzing user-generated content. Text-mining large corpuses of readily available data can reveal subtle, often overlooked factors that can influence positive or negative aspects of user experience, providing a comprehensive understanding of satisfaction and dissatisfaction.

Another powerful method is HVM (Gengler et al., 1995), which visually represents the relationships between product attributes, their consequences, and the ultimate values sought by customers. This approach has been used in contexts ranging from hospital experiences (Kumar et al., 2020) to perceptions of autonomous vehicles (Merfeld et al., 2019), illustrating its versatility in identifying and addressing key satisfaction and dissatisfaction factors.

Once these contextual factors are identified, understanding their varying impacts becomes crucial. Different factors influence (dis)satisfaction in distinct ways, often varying by domain. For instance, in e-learning platforms, the quality of the system might drive satisfaction, while responsiveness to user needs might have a greater impact on dissatisfaction (N. Um, 2021). Tailored measurement tools, such as domain-specific unipolar scales, allow for existing idiosyncratic differences to be captured effectively. Structured approaches like the House of Quality (HoQ) can help translate customer requirements into actionable insights, prioritizing areas for improvement (Hauser, 1993). Additionally, using a threshold-based analysis within ESG zones helps identify specific levels of (dis)satisfaction that correspond to meaningful decisions or behaviors.

### Separate measurement of separable unipolar constructs

The debate over whether bipolar or unipolar scales are more effective for measuring attitudes and opinions remains ongoing (see Alwin, 2007, 2010; DeCastellarnau, 2018; Höhne et al., 2022; Krosnick and Fabrigar, 1997; Menold, 2019; Menold and Raykov, 2016; Schaeffer and Presser, 2003; Thomas and Barlas, 2018). However, when ambivalent states are likely, unipolar measurements are essential. Evidence from multiple domains suggests that (dis)satisfaction are distinct constructs arising from different factors, necessitating independent measurement with multidimensional scales.

Unipolar scales allow for a more complete understanding of coexisting phenomena, where both positive and negative feelings may be present. Unlike single-item measures, which oversimplify attitudes, multiple-item scales provide richer insights into complex scenarios (Gliem and Gliem, 2003; Nunnally and Bernstein, 1994). For example, while a single unipolar item measuring drug liking may correlate with a bipolar item (*r* = 0.72; Setnik et al., 2017), collecting unipolar items for both liking and disliking reveals more actionable insights, especially in cases of ambivalence (e.g., short-term liking versus long-term disliking of the drug’s effects).

Adhering to proper scale design practices is also crucial. Schrum et al.’ (2023) reviewed the human-robot interaction literature and found that only 4 of 144 adhered to best statistical and design practices when using Likert scales. This underscores the challenges of constructing robust multidimensional scales, particularly for domains where bipolar interpretations oversimplify complex constructs. Rather than critiquing specific areas, we aim to improve measurement and interpretation practices by advocating for the appropriate use of unipolar scales. We echo Schrum and colleagues’ sentiment in their Nota Bene (p. 3) in that we have not always employed best practices in our prior work, and that we only seek to call out that unipolar scales might be useful when simplified bipolar interpretations needlessly reduce the complexity of the overall picture.

#### Use asymmetrical response options

To measure (dis)satisfaction independently, asymmetrical options are highly effective. They reflect the reality that dissatisfaction often carries greater psychological weight than satisfaction—a phenomenon attributed to the negativity bias. For instance, dissatisfaction with a service can prompt immediate discontinuation, while satisfaction may merely sustain the status quo. Asymmetrical scales with detailed gradations for dissatisfaction can capture subtle variations in negative experiences, revealing when dissatisfaction crosses thresholds that trigger complaints or switching behavior.

#### Threshold-based analysis using ESG zones: expanded approach for ambivalent and unipolar measures

Threshold-based analysis within the ESG provides a robust method for interpreting unipolar scale data. ESG zones—polarized positive, polarized negative, indifferent, ambivalent, and neutral—capture specific levels of (dis)satisfaction that correlate with meaningful decisions or behaviors. For example, the ambivalent zone captures the complexity of customer satisfaction by addressing both positive and negative evaluations simultaneously, a concept crucial in refining midpoints that otherwise might obscure insights due to respondents’ mixed emotions (Audrezet and Parguel, 2018; Yu and Davis, 2024). We next detail these thresholds:

##### High satisfaction threshold

Responses that fall in the lower-right zone represent high satisfaction with minimum negativity, suggesting loyalty and a likelihood of repurchase or recommendation.

##### High dissatisfaction threshold

Responses in the upper-left zone of the ESG indicate high negativity with little positivity, where respondents may complain or abandon a product. Understanding these thresholds can help identify areas requiring immediate intervention to prevent customer loss.

##### Indifferent-to-neutral zone

The bottom-left zone of the ESG reflects low positive and negative activation, signaling indifference. This state presents opportunities for targeted strategies to enhance engagement and satisfaction.

##### Ambivalent zone

Captured using the upper-right zone of the ESG are strong, conflicting emotions. Insights from this zone can help resolve negatives and reinforce positives by prioritizing improvements in critical areas, such as service quality or delivery issues, while highlighting strengths.

#### Advantages to threshold-based measurement

Using thresholds derived from the ESG enhances diagnostic precision by segmenting responses into distinct levels of satisfaction and dissatisfaction. This approach captures critical shifts in sentiment, allowing stakeholders to act on both positive and negative feedback. By refining midpoints and measuring the coexistence of emotional responses, researchers can obtain a comprehensive understanding of customer sentiment, enabling more effective strategies for improving satisfaction and addressing dissatisfaction.

## Dynamic final thoughts

In interpreting satisfaction and dissatisfaction as distinct, unipolar constructs, we open the door to a more flexible understanding of human emotions, motivations, and decision-making processes. The traditional bipolar framework, which posits that satisfaction and dissatisfaction are mere opposites, simplifies a complex reality and can obscure critical insights into how people navigate ambivalent or indifferent emotional states. By recognizing that (dis)satisfaction can co-exist, and that each is driven by separate factors, we are better positioned to capture the full spectrum of human experience.

Recognizing the unipolar view’s utility to not only enrich our theoretical understanding but also pave the way for innovative methodological approaches, such as dynamical systems modeling. By applying dynamical systems theory to the study of (dis)satisfaction, we acknowledge these emotional states as dynamic processes that evolve over time in response to various internal and external influences. This perspective allows us to capture the complexities and fluctuations inherent in human experiences, offering a more holistic understanding of how (dis)satisfaction develop, interact, and influence behavior over time.

Dynamical system modeling enables researchers to visualize and analyze the temporal patterns and feedback loops between (dis)satisfaction. For example, initial dissatisfaction with a product feature may lead to decreased usage, which further exacerbates dissatisfaction—a negative feedback loop. Conversely, positive experiences can increase engagement, creating a positive feedback loop that enhances overall satisfaction. By identifying these dynamic interactions and critical thresholds where significant shifts occur, we can develop more effective strategies to enhance satisfaction and mitigate dissatisfaction before they become entrenched.

In conclusion, embracing the bivariate unipolar view of (dis)satisfaction provides a powerful framework for understanding complex emotional states, motivations, and behaviors across various fields. By moving beyond simplistic bipolar scales, especially in ambivalent situations, we can better capture the intricacies of human experience, ultimately leading to improved measurement, deeper insights, and more effective strategies for enhancing satisfaction while mitigating dissatisfaction over time. This dynamic approach offers a richer, more flexible understanding of the interplay between positive and negative experiences in shaping our decisions and actions, recognizing that these processes are not static but evolve throughout our interactions with products, services, and experiences.
